# A Descriptive Qualitative Study of the Perceptions of Regulatory Authorities, Parents, and School Canteen Owners in the South of Ecuador about the Challenges and Facilities Related to Compliance with the National Regulation for School Canteens

**DOI:** 10.3390/ijerph20075313

**Published:** 2023-03-29

**Authors:** Belén Cabrera-Ledesma, Victoria Abril-Ulloa, Verónica Pinos-Vélez, Valeria Carpio-Arias

**Affiliations:** 1 Faculty of Medical Sciences, University of Cuenca, Cuenca 010201, Ecuador; 2 Research Group: “Public Health, Food and Physical Activity in the Life Cycle” Career of Nutrition and Dietetic, Medical Sciences Faculty, University of Cuenca, Cuenca 010201, Ecuador; 3 Chemical Sciences Faculty, University of Cuenca, Cuenca 010201, Ecuador; 4 Research Group GIANH, School of Nutrition and Dietetics, Faculty of Public Health, Escuela Superior Politécnica de Chimborazo, Riobamba 060155, Ecuador

**Keywords:** challenges to promote healthy eating, school canteens, healthy food, food, processed and ultra-processed products

## Abstract

The prevalence of overweight and obesity in the Ecuadorian school population continues to increase. An important factor in children’s nutrition is the food offered in school canteens. The objective of this study was to explore the perceptions of the challenges faced by and facilities of school canteens in the provinces of southern Ecuador in terms of complying with national regulations. For this qualitative descriptive study, semi-structured interviews were conducted in 2021 with six school canteen owners, six school directors, five health experts, and seven parents of children attending school from three provinces, Cañar, Azuay, and Morona Santiago, in Ecuador. The data were transcribed and subsequently analyzed in ATLAS ti. The participants indicated several challenges to comply with the regulations of school canteens, such as the expenses generated by them, the lack of control of street food vendors in the surroundings of the schools, and the lack of trained personnel. Regarding the facilities, they highlighted that the regulation for cleaning and hygiene are more easily fulfilled. Standards and control of the food stipend in school canteens are required to ensure a supply of healthy food for the children. Multiple challenges and strategies are proposed to improve the eating habits of the school population and to improve the nutrition of schoolchildren.

## 1. Introduction

Obesity is a public health problem that affects the entire population and continues to worsen. In the last 40 years, the global prevalence of overweight and obesity in children and adolescents aged 5–19 years has increased more than fourfold, from 4% to 18%. The majority of overweight children live in developing countries [1]. In Ecuador in 2018, it was reported that 35.4% of children and 29.6% of adolescents are overweight and obese [2]. Food and physical activity are factors related to the development of overweight and obesity, and, in addition, home and school are recognized as important environments for promoting healthy habits [3].

The Food and Agriculture Organization of the United Nations indicates that holistic and coherent school programs and policies are essential for the fulfillment of children’s rights to food, education, and health. Programs that promote healthy eating and nutrition education will enable children to improve their diets, acquire and develop healthy eating habits, and extend them to their families and communities. In addition, regulations and policies are required to promote the generation of healthy environments that favor the availability of pleasant, safe, and nutritious foods to improve school feeding. Healthy environments include school facilities where food is purchased or consumed, such as shops, kiosks, canteens, etc., in addition to food prices and food availability [4]. Some countries have implemented several public policies that include the provision of healthful foods/beverages, fruits, vegetables, quality standards for school meals, and others. Following evaluation, the impact of these policies is unclear, and some studies have focused on determining the dietary composition of food intake at school and in the homes of students and found that these policies improve the intake of fruits and vegetables while reducing sodium intake. However, the differences across countries could lead to varying results [5]. On the other hand, determining the facilities or barriers for school staff implementing changes at schools is little studied. A study in the United States evaluating the perceptions and experiences of school staff and the school food environment after building renovation to support healthy eating found that the preferences of children improved due to the exposure to healthy foods [6].

Moreover, it is important consider the concept of the food system, which includes the entire process from production to the consumer [7]. In this sense, school canteens are a focal point, and could offer foods that contribute to whether children have healthy or unhealthy diets. Moreover, considering that ultra-processed foods can be cheaper than healthy foods [7], the public policies of the Ecuadorian state are focused on eradicating malnutrition via the promotion of healthy eating and the balanced consumption of nutritious and healthy foods, which are typical of the region, and other aspects that guarantee adequate nutrition. In 2014, the regulation for controlling the operation of school canteens in the national education system focused on ensuring that food and drinks sold in canteens must be innocuous and contribute to a varied and healthy diet for students [8].

Although the generation of public policies focused on promoting healthy eating is important, it is also important to know how they have been implemented in schools and educational centers, and to evaluate perceptions of the challenges and of the facilities related to their execution. The objective of this study was to explore perception of the challenges and facilities by the owners of school canteens, directors of schools, health experts, and parents regarding compliance with the regulations of school canteens in the provinces of Azuay, Cañar, and Morona Santiago in southern Ecuador.

## 2. Material and Methods

### 2.1. Study Design

This is a descriptive qualitative study carried out according to [9], which will allow investigating and understanding of the perceptions regarding the challenges and facilities of some school canteen owners, school directors, health experts, and parents according to their experiences related to the implementation and control of compliance with the operating regulations for school canteens. The qualitative description allows for obtaining answers about the emotions and perceptions that the people involved with the services or functions of space have, in this case, the school canteens that are governed by the national regulation in Ecuador. This manuscript was prepared according to the Consolidated Criteria for Reporting Qualitative Research (COREQ) [10].

### 2.2. Context

In 2014, a regulation was issued in Ecuador to control the operation of school canteens in the national education system, which is focused on establishing requirements for the administration, control, and proper operation of school canteens, where the preparation of innocuous and nutritious food is contemplated, in addition to promoting healthy eating practices and health in educational institutions [8]. Since this regulation was issued in Ecuador, several actors related to school feeding have had various experiences related to its implementation and compliance control. Depending on the work and activities related to the operation of school canteens, the experiences of the participants are diverse, and it is necessary to characterize them to identify challenges and facilities that participants have experienced, which can serve as evidence for possible changes or improvements that could be made in the regulation in question and their necessity. The regulation of school canteens includes establishments within educational centers that sell food to children and adolescents from 6 to 18 years of age. In this study, semi-structured interviews were conducted with various actors from three provinces in southern Ecuador during the year 2021. Although the schools were closed at the time of the interviews due to COVID-19, the participants were asked about their experiences when the schools and school canteens were operating before the pandemic.

### 2.3. Participants

The list of owners of school canteens and directors of educational units was requested from the zonal Health Coordination 6, and one of the researchers of this study (BCL) contacted the participants by telephone: six owners of school canteens, six directors of educational units, five health experts, and seven parents of children from 6 to 11 years old agreed to participate and received comprehensive theoretical information about the study. In the initial contact with the participants, 4 people did not agree to participate in the study and 12 did not answer.

### 2.4. Collection and Analysis of Information

The interviews were conducted and recorded from April to June 2021 by video call on the Zoom platform, with prior authorization from the participant. Each of the interviews lasted approximately 45 min. Prior to conducting the interviews, a small pilot test was carried out to validate the understanding of the questions. Verbatim transcription of the recordings was carried out, and categories were determined for analysis of the information, which was carried out using the ATLAS ti ^®®^ program. The interview questions were focused on the following questions: challenges and facilities regarding compliance with the school canteen regulations, and the sale price of school canteen products. The information analysis was carried out as a thematic analysis through an inductive coding process in reviewing the transcripts to create codes and subcodes.

The process of analysis follows the phases described by Braun and Clark: familiarizing yourself with the data; generating initial codes; searching for themes; reviewing themes; defining and naming themes; and producing the report [11].

To protect the identity of participants, we assigned them a number for participation in the study given to experts, parents, owners of school canteens. The health experts are referred to by their initials (Table 1).

### 2.5. Rigor

The data collection process of the research is described in detail. The interpretation of results was supported by the triangulation of investigators. Two independent researchers reviewed the analysis and results of the obtained information. The participants and the study setting are described in detail.

### 2.6. Ethical Aspects

This project was approved by the Research Bioethics Committee of the Health Area of the University of Cuenca with the number 2020-288EO-MST-EP. Prior to conducting the interviews, all participants signed an informed consent form, where the objective, methods, risks, and benefits of their participation in the study were explained. The consent was sent by mail and to one of the researchers of the study in digital format. The study was carried out under the considerations of the Declaration of Helsinki.

## 3. Results

Of the 24 participants, there were 10 males and 14 females corresponding to 5 health experts, 6 school canteen owners, 7 parents, and 6 school directors that were interviewed. Their characteristics are described in Table 1. From the inductive thematic analysis, the following themes and subthemes emerged: challenges to compliance with the school canteen regulations (subtopics: expenses generated by the school canteen that make it difficult to sell healthy food, motivation of healthy food consumption at home and in schools, functions performed by the control committees and other detailed in this section.); facilities for compliance with school canteen regulations (cleaning of the school canteen and use of uniforms); and cost of healthy food.

### 3.1. Challenges to Compliance with School Canteen Regulations

According to what all the groups of participants indicated, there are several challenges in compliance with the school canteen regulations.

Expenses generated by operation of the school canteen. The operation of the school canteen generates expenses that must be covered by the owner of the school canteen. Among these are the payment of rent and the hiring of personnel, in addition to covering expenses, before having profits. On the other hand, usually the most attractive products to be bought by children are those that are not healthy. In this way, all participants consider the offer of high-calorie foods in school canteens to be justified due to the above mentioned challenges, which may interfere with compliance with the regulations.

On the other hand, the owners of the canteens indicated that they intend to sell healthy food and have healthy food available, but that these are not attractive to students, which generates economic losses.

Health experts, parents, and directors of schools agreed that one of the most important obstacles to promoting healthy foods for students is the products sold in school canteens. Moreover, there is evidence that the environment is a determinant of nutritional habits [12].

*“We want to sell nutritious food, but the children don’t buy it. It is not convenient for us to prepare something that they do not buy, even if it is nutritious. We must pay the rent and the people who work in the school canteen, and if we don’t sell products that catch the attention of the children, we lose the sale because they are going to be bought elsewhere.”*

Owner4

*“School canteen owners see the canteen as a business, not a service. The authorities can make regulations, but they remain on paper, in practice, they are not executed. School canteen owners work for financial gain. Generally, what is nutritious, what is healthy, is not always what generates economic resources; therefore, the sale of healthy food is complex.”*

Director1

*“In school canteens, healthy food is rarely sold. So, I don’t know if the strategy is working. I have seen the students eating sandwiches with mayonnaise, potato chips in bags (processed), and things like that.”*

Parent4

Motivation for food consumption at home and in school. Health experts, parents, and directors of the educational units expressed that the purchasing preferences and food consumption of unhealthy products by children are barriers to compliance with the national regulation.

They agree that, in some cases, children do not eat healthy food at home and do not know if the food or products that they consume are healthy. Even parents send these products with children to schools. In other cases, the teachers at the schools promote the preparation of unhealthy foods, and this is observed and experienced by the children. The majority of participants agreed that it is important to educate children in school and that the practice of consuming healthy foods at home should be reinforced, generating healthy environments with the example of school authorities, parents, and teachers, which would allow that learning to have a long-term impact.

*“Children will not consume healthy food if the example is not set, many of the children’s caregivers are overweight or obese and even if they talk about healthy eating at home, if they do not show by example, it is not feasible for children to eat healthy because it is like talking about a subject without knowing it.”*

Expert4

*“I believe that what we should do is try to teach children how to eat and that does go from home, but children also spend a lot of time in schools that are like their second home, so they should also be educated there about healthy eating.”*

Parent6

*“Children often take warnings about unhealthy eating lightly and are not aware that something is going to happen to them, so they should teach them how to eat at home. A nutrition subject should be included in school so that they learn and know how it affects food.”*

Owner6

Functions exercised by the control committees: Among the health experts, there seems to be confusion about the tasks that fall to each institution to control school canteens and ensure compliance with the regulation. They state that there is no empowerment of all those who make up the School Canteens Committee (comprising one delegate from the Ministry of Health, one from the Ministry of Education, and one from ARCSA) and indicate that it would be necessary for the Ministry of Education to collaborate and adopt a more active role for compliance with the regulations, and that food be appropriate in educational units.

This suggests the need for additional standards and protocols for the operation of these committees.

On the other hand, the owners of school canteens and directors of schools declare that the health experts who carry out the control of school canteens have different criteria regarding the food that can be prepared and sold in these establishments since they allow them to occasionally sell fritters on some occasions, which is contrary to what is indicated in the School Canteen Regulations.

*“The institutional committees are formed but do not fulfill their functions.”*

Expert1

A worrying situation is that some health experts indicated that the supervisions in the canteens are not the same, and that they do not even use the same forms to control compliance with the regulations.

On the other hand, the directors of schools feel, in some way, that when monitoring is carried out, it does not go beyond the reports and recommendations and that no changes result. They also indicate that it is necessary to have sanctions for those who do not comply with the regulations of school canteens.

*“Committee messages are not clear and can be contradictory. For example, we are not sure what to do with the frying oil, whether we can reuse it or not.”*

Owner4

*“The owner of the school canteen was allowed to sell a fried food such as salchipapas (French fries with sausage) once a month, although it is not allowed, but as an institution, we let it because we consider that once a month there is nothing wrong with it, the problem would be eating too many fried foods.”*

Director3

Lack of control of street vendors around the schools: All groups of participants stated that there is a lack of control of the street vendors around the schools. This generates discomfort in the owners of the school canteens since the control of the sale of products and food is made only inside the schools. They perceive that it is not fair to only control the school canteens while there is no type of control outside the units. This allows children to have access to unhealthy products and foods and promotes non-compliance in the sale of healthy foods in school canteens as the owners seek to obtain an economic benefit from the sale of their products.

*“Street vendors are outside the school. The kids can’t go out, but the street vendors do things like throw products and money over the wall to sell to the kids. The people from the Ministry of Public Health tried to close the spaces where the vendors passed the food, but that is not the solution.”*

Director1

*“The regularization of street vendors should be the responsibility of several authorities. But nobody regulates it.”*

Expert5

*“The children like the food that the street vendors sell. But there is no control with them.”*

Parents5

Lack of collaboration and responsibility on the part of school canteen owners: Some health experts and parents consider that there is little collaboration from some school canteen owners, since they try to have the right food when supervision or monitoring is carried out, but they do not maintain selling these foods all the time. On the other hand, any call for attention made by health experts in the face of non-compliance with the regulations is not positively received by the owners of school canteens.

*“Canteen owners know the rules, but they know which foods sell the most (assuming they sell foods high in fat, sugar, and sodium).”*

Parent4

*“School canteen owners can be very aggressive when we visit the school canteens.”*

Expert4

*“For the owners of the canteens it is a problem that, suddenly, other people sell food.”*

Owner3

Marketing and promotion of food and food products: Health experts, parents, and directors of schools indicate that the marketing of unhealthy foods through different advertising media and social networks influences the consumption of these products in children. They consider that carrying out advertising campaigns for healthy foods and healthy food education could change children’s habits, and thus, begin to select healthy foods to buy and consume.

*“The factor that most affects children’s nutrition is the media and advertising.”*

Director4

*“We are in a new digital age. Television, and social networks promote food products. It would be very interesting to start doing healthy advertising. The media tries to convince people that some athletes consume soft drinks.”*

Expert4

*“Homemade ice creams cannot compete with branded ice creams, because of advertising”*

Parent3

Little variety of food in school canteens: Health experts and parents suggest that is necessary to offer more attractive, varied, and healthy foods since the options usually are limited. However, the owners of school canteens and school directors state that when something different is done, they do not receive much reception from the students and the food is damaged.

*“Before, we used to make lasagna with sweet bananas We made hot dogs, without mayonnaise. We made vinaigrettes instead of mayonnaise, but little was sold.”*

Owner2

*“The food guides or list for foods that are expended in school canteens do not have many alternatives. More creativity is needed.”*

Expert1

*“It is necessary to have more variety of healthy food.”*

Parent3

Choosing unhealthy foods according to tastes and preferences: Parents, owners of school canteens, and directors of educational units believe that many children buy and eat unhealthy foods because they like the taste and prefer to eat them instead of healthy foods. Even if they are prohibited from consuming them and they know that it is not healthy, they will consume them. On the other hand, there is also the issue that some parents give their children money to buy food at school and the children will prefer to buy the food they like.

*“The most influential factor in choosing a food is its taste.”*

Expert5

*“Food preferences is a very important topic. The students prefer to eat hot dogs instead to eat fruit salad. They do it because of their smell or because of the influence of their peers.”*

Parent4

*“Students don’t taste nutritious food; they say the taste is ugly. I tell them to try it and not pay me, but they say they don’t even want it for free.”*

Owner4

Need for professionals in food, nutrition, and gastronomy: Health experts, parents, and directors of schools agree on the need to have nutritionists and gastronomy professionals in schools to improve children’s nutrition and guide them in providing adequate portions.

*“should be in charge a staff that is already professional in the field of gastronomy and has a lot of knowledge in the field of preparations.”*

Expert2

*“I think it would be good that, just as there are psychologists in schools, doctors and others, there should be a top nutritionist because they can guide the amount, even the portion that children could or would consume.”*

Parent6

*“The portions are subject to everything that the nutritionist says, so the nutritionist arrives at any time and asks what she is doing. They look at the portions, the weight, the carbohydrates, what they should have, a healthy snack or a healthy lunch, they are in charge of seeing the measures, what exact amount students need.”*

Director4

The infrastructure of the school canteen: Health experts and one of the directors indicate that the infrastructure is inadequate. They state that the Ministry of Education is responsible for investing the proceeds from the rental of the canteen to carry out these improvements; however, this is not being fulfilled.

*“Many times… the canteen does not comply with the adequate infrastructure, the spaces are very small, many times they are very open, and this depends on the educational unit, not only on the administrator, the same agreement tells us that everything collected from the rent of the canteen has to be arranged for readjustments of the same which is not being fulfilled.”*

Expert2

*“I don’t know if it depends on the school canteens, but the infrastructure is super important to improve. The place is for the children to sit down to eat. They have some little tables so that they really enjoy themselves because they also eat quickly. Even if it is healthy, it is not good for them.”*

Parent5

*“They told me that I had to improve the infrastructure of the canteen, which was impossible because it was something leased. I received the facilities as they were given to me, and that is how I had to maintain them myself. I made an expense there that the institution never recognized me for, I had to change the roof, I did some electrical installations and other improvements.”*

Owner5

Control of the food prepared at the canteen: Health experts and parents agree that the control has focused on the elimination of industrial packaged products and not on food prepared in school canteens. The concept of ultra-processed is confused with industrial or homemade, and attention to the content of the food is lost. For example, it is common to find foods high in sugars and fats prepared in the same canteen without any control over nutritional content or compliance with hygiene standards. For this reason, there is a consensus in suggesting greater control of the preparations made in the canteen.

*“They can prepare any food and if it is high in fat or sugar we cannot say no because the prohibition is for the sale of processed and ultra-processed foods, and with that, we cannot do anything at all, that is contradictory, we do not have a way to carry out an exhaustive control let’s say for the amount of fat and sugars that they put in their preparations.”*

Expert2

*“my children come home and say: Mommy today we ate carrot cake, supposedly a healthier snack, but it is a cake with a lot of cream full of sugar. Another day he told me: today they gave us an ice cream, he still said that they are cream and they are still sweet, so that should be controlled in how they do it.”*

Parent3

*“They had not been processed, but there were bolones (fried plantain with cheese or pork rind), Cubanos (sandwich with cheese, ham, mayonnaise, tomato and lettuce), pork sandwiches, chicken sandwiches all with mayonnaise, hamburgers, hot dogs, pizzas, jellies, if you see that nothing is covered, but in the end, in my opinion, they are not healthy.”*

Director6

Need for sanctions for non-compliance: The participants believed that the lack of or inadequate sanctions for non-compliance with the norm means that they are not adequately applied in practice. In addition, greater control over the preparations made at the canteen is suggested. The study groups consider that a solution to ensuring compliance to the norms by the owners of the canteens is the application of a fine.

*“Characteristics should be stipulated are the sanctions with reference to nutrition …unfortunately I think that this way they become more aware, we say that for an operating permit they must pay five basic salaries and for hygienic sanitary conditions they must pay 10 salaries basic, while for the nutritional field there is no type of sanction clearly it is said that the sale is prohibited but there is nothing that sanctions or that should be done in terms of non-compliance.”*

Expert3

*“But if there are administrators who, no matter how much they control them, the next day they sell again because they know that those from ARCSA are not going to go two days in a row and if they take advantage of it because we have no limits in what we can prepare. There is no article that directly sanctions administrators.”*

Owner5

*“The gentlemen must be sanctioned because they care little or the same, they do not improve despite the calls for attention and it is not only from the directors and the food commission since they are general meetings with all the representatives of 1200 children, but no, if there is no sanction they do not improve.”*

Director2

### 3.2. Facilities for Compliance with School Canteen Regulations

According to health experts, it is easy to check the labels of processed products in complying with the regulation. According to most participants, it is easier to comply with the provisions of the regulations in terms of cleanliness and proper handling of food.

Cleanliness of the school canteen and use of uniform: Parents, directors of schools, and owners of school canteens agree that the application of the norm has improved the cleanliness of canteens, and they see the implementation of the use of uniform as an advancement; however, the health experts indicate that there are things to improve. For example, in their monitoring, they have verified the simultaneous handling of money and food, in breach of the school canteen regulations.

*“Cleaning, dishes… removing the packaged products that have been requested, that is easy.”*

Owner5

*“I don’t know if it’s part of the regulation, …they’re constantly cleaning it so it’s always clean…”*

Parent4

*“the easiest thing to accomplish for them is cleaning. It is not difficult for them to keep everything clean because that is an image for those who visit the institution. It is something that would attract attention if it was not presentable, so they each sometimes they sweep, clean, cover the food, they are very interested in that care.”*

Director5

### 3.3. Cost of Healthy Eating

The price of food emerged as a reason for choosing food. However, there is no consensus on this because while parents say that eating healthy is much more expensive than eating less nutritious foods, the latter is chosen, and the remaining groups have divided opinions; for example, the health experts and the directors of the educational units believe that it is partly the cause of the problem, but that it is not the greatest obstacle in their choice. In another example, the health experts and directors of the schools indicate that the so-called school snack, which is a free and healthy breakfast financed by the state, was not consumed by most children.

*“For example, a bottle of water that costs 50 cents. They went outside and bought a bottle of soft drink for 25 cents. We sold the juice for 25 cents, and they already told me that outside the sodas have the same price and that it is tastier, so they prefer to go there to buy because they say that more is coming.”*

Owner5

*“It is that not only can it be that just for wanting something healthy we only buy fruit and that’s it, it is not enough. If I offer sandwiches, I would try to make it healthier, but that is already more expensive if I want the sandwiches to put a cheese healthy low-fat a whole meal bread, if it could be improved, but it is more expensive.”*

Parents6

*“Generally, a processed food is much cheaper than a food that is prepared in the school cafeteria. This is firstly because of the ingredients that are used and then because, logically, in an industry it is done commercially and on a large scale. So healthy food is more expensive than unhealthy food, processed food is much cheaper than any other food.”*

Expert3

## 4. Discussion

School environments are important to promote healthy eating, which could reduce childhood obesity. Some countries have created programs and developed policies to promote healthy lifestyles. Schools that offer opportunities for physical activity and access to healthy foods are associated with engagement in health behaviors and improved learning and academic performance, particularly among children from low socioeconomic status households [13]. The sale of unhealthy foods within schools promotes unhealthy dietary behaviors through high intake of sodium and sugar, and inadequate intake of fruits and vegetables [14].

This study contributes to the perspectives of different actors on the facilities and challenges for compliance with the Ministerial Agreement of school canteens [8] in the southern zone of Ecuador in relation to their operation.

As in many countries, the sale of food in school canteens in Ecuador is regulated by government agencies [15]. The country’s public policy bases its actions on the control of the availability of unhealthy foods as one of the strategies to improve the health of the population. These policies are also framed in need to carry out hygienic and sanitary controls of food dispensing. In this study, it was found that public policies for the operation of school canteens do not work as they should. Thus, non-compliance with the regulations was indicated by the owners of the canteens in addition to a lack of support related to clear messages about the procedures that must be followed. An example of a healthy school canteen experience could be the initiatives carried out in the United States, where school canteens that sell fruits and vegetables have been implemented [16].

In this study, the main actors—parents, directors of schools, and health experts—agree that many school canteen owners do not comply with Ministerial Agreement 005-14 because the sale of unhealthy foods has not been completely eliminated. It is also mentioned that canteen owners do not abide by the provisions due to a lack of sanctions regarding the open preparation of artisanal products. This suggests that more control actions and constant monitoring should be carried out in the canteens. This reflection leads us to think about the consumption of processed foods in children and the strong influences of market and advertising systems. Therefore, it is suggested that public policies for the sale of food be reinforced [17]. Moreover, education and knowledge in healthy eating, for example in the consumption of fruit, and its importance is necessary [17].

On the other hand, in this study, it was found that the sale of artisan foods, and their preparation and ingredients, should also be monitored; although they are considered natural, their nutritional composition is not always the most appropriate. This allows us to confirm, again, the importance of nutritional education in the population [18].

Healthy foods should be creatively displayed and placed in such a way that they are the first choice of schoolchildren. When preparing food, sauces or creams should not be added, which has been a challenge for school canteen owners. Parents and owners of school canteens state that wanting to make food with more purely nutritious options involves a greater economic expense. Healthy eating has been considered today as an expensive diet, which leads us to think about the challenges faced by both canteen owners and consumers. Health experts and the directors have a divided opinion on the matter, but some studies seem to affirm that a healthier diet is more expensive and has its disadvantages; for example, a study carried out in Ontario, Canada, shows that nutritious foods are more expensive than healthy foods [19]. However, in a study in Mexico, no differences were observed between the costs of a healthy and a less healthy diet [20]. On the other hand, it could be interesting to consider the money that parents give to their children to buy food in schools and the differences by socioeconomical level, or in private or public schools.

Most of the school canteens have reduced the sale of processed and ultra-processed products that are not allowed, but they lack healthy alternatives. Marketing and other strategies or observations must be considered. The Internet is now a major platform for food marketing, and a study found that influencer marketing of unhealthy food promotes children’s intake of unhealthy food [21].

Several countries have implemented actions focused on reducing the consumption of unhealthy foods, such as introducing taxes, reducing the availability of these foods in schools, marketing restrictions, ad labeling, among others; however, this does not prevent children consuming products with high energy density before or after attending school. On the other hand, although the regulations are focused on reducing or eliminating the sale of processed and ultra-processed products, is important consider that some foods made in school canteens may contain high amounts of sugar, fat, and salt, the consumption of which is related to overweight, obesity, and chronic diseases, such as diabetes and cardiovascular diseases particularly affecting the low-income people of Latin American countries [6,22].

Parents, directors of schools, and owners of school canteens perceive that compliance with the regulation and its intention to promote healthy eating is not working, because the consumption preferences of students are not changed. Canteen owners indicate that their initiatives to sell healthy food have failed because children seek alternatives outside of school to obtain food from which they are restricted to sell, which causes them economic losses. For this reason, given the impossibility of selling industrial packaged foods, the canteen owners compensate for this demand themselves by cooking unhealthy foods, such as fried foods and sweets, that are desired by the students. This shows that despite the good intentions of the standard, it is not working.

Regarding intervention programs focused on restricting access to caloric foods for children and adolescents, studies worldwide do not show sufficient evidence of having achieved changes in food consumption habits [23]. Within community settings, mixed impacts have been found when using strategies, such as economic incentives, restrictions on the commercialization, and marketing of foods with a high fat and/or sugar content, implementation of labeling, interventions to advise food choices, implementation of diets, and marketing strategies, among others [24,25,26]. The only example for which consistent evidence has been found is that providing toys with meals increases their selection by children [27]. Studies conclude that despite finding small improvements in diet and nutrition outcomes associated with many of the examined interventions, implementation of individual interventions has little impact on diets and nutrition [27]. Due to the aforementioned, results will only be achieved with interventions that achieve a permanent change in preferences and consumption habits; the achievement of both is not easy, but is essential. To achieve this, interventions should focus not solely on schools, but extend to the community with an emphasis on families, and this should include the food supply chain; only in this way can the supply of healthy, affordable, and attractive food be guaranteed in children’s environments [28]. In school, it could be helpful to carry out healthy marketing, conduct campaigns, place posters in schools, ensure that teaching is continuous and that there is an explanation in the classrooms about the nutrients contained in the products sold at the canteen, and that there is also involvement on the part of the student in cases where this target population is not actively participating in achieving a healthy diet. At home, parents should promote healthy environments with nutritious food and physical activity.

Changing preferences and eating habits in children is essential. The Food and Agriculture Organization of the United Nations (FAO) has established that children must be considered the adult consumers of tomorrow. Food customs are learned early, and educational institutions can play an important role in promoting healthy and sustainable food selection criteria and dietary guidelines. However, it is unreasonable to expect children to change their eating habits without the rest of the population doing the same. The FAO encourages the implementation of training programs that make the population aware of their rights in terms of food, adequate diets, food safety, foodborne diseases, labeling, and processing, among others [29]. In other words, the entire population should be trained and involved in decisions regarding food, and thus, create a healthier environment for children. The programs must be varied and not only focused on presenting the nutrients and properties of food since studies indicate that these programs are useful for children in learning to identify foods and their nutritional properties, but not in changing habits. For training-type interventions to generate changes in addition to health, they must be connected to taste and social experience, such as familiarity [30].

In fact, although educational programs focused on healthy eating are necessary, in practice different factors intervene in a child’s choice of food, among which taste stands out [31]. For example, studies have shown that a preference for healthy food is related more to the marketing of more varieties of palatable healthy foods than to eliminating other types of food [32,33,34]. Therefore, along with regulating the nutritional quality of food, offering healthy foods that are also appetizing should be encouraged. The experience and example of consumption are other important factors in promoting a healthy diet. This study indicates that children are expected to make correct decisions regarding food when they do not see that example at home and or even at school, where there are teachers who frequently allow themselves to consume foods with high fat and sugar content. With these examples, the importance of culture and family is evident since when food that is traditionally consumed in a community is not healthy, this will be reflected in consumption preferences.

The sale of unhealthy foods within schools promotes unhealthy dietary behaviors among children, and the lack of infrastructure, human resources, and the cost of healthy foods could moreover be an impediment to compliance with policies to promote healthy eating [14].

This study explores perceptions about the challenges and facilities of school canteens in Ecuador regarding implementing the ministerial agreement. It is the first contribution in this sense, and this information may be useful for other studies and for the analysis of stakeholders toward improving the current situation of the sale of products in school canteens.

Among the limitations of this study are that being a qualitative study, it does not allow the extrapolation of the information to the national territory. In addition, the information was taken during the time when schools were closed due to the COVID-19 pandemic. This may result in some biases in the collection of information since only those participants who have access to the Internet can carry out the interview. There are also people who are afraid of speaking in front of a camera, and this could influence some responses.

## 5. Conclusions

The implementation of public policies to promote healthy eating is a challenge considering those involved in their control and execution despite having a predisposition to the school environment, and economic factors can be a complex barrier to overcome.

School canteens generally offer unhealthy food and products characterized by high contents of salt, fat, or sugar and, on the other hand, little supply of fruits and vegetables. Parents, school canteen owners, and school directors consider that schools are suitable places to promote healthy eating, and that including food and health education in the curricula can be very useful such that children are the ones who consciously want to eat healthily. On the other hand, the importance of children being in an environment where good habits are promoted at home and at school is emphasized, where parents and teachers are examples of good habits and healthy lifestyles; otherwise, it will be very difficult for only educational content to exert a change in children’s behavior.

In addition, it is important that the food offered in canteens is pleasant, not only in taste, but also visually because children prefer those foods that they find pleasant.

Another important aspect is the cost of healthy foods, which are often considered to be more expensive than foods with no nutritional value.

Among the suggestions given by the participants to comply with the regulations is the execution of sanctions for those who do not comply with what is established in the regulation, including qualified personnel in nutrition and gastronomy in the school canteen that prepares healthy, tasty food that is also in accordance with the food culture of the region, and the inclusion of foods that are low-cost, but nutritious and healthy.

Correctly executing these actions to promote healthy environments could contribute to the reduction not only in overweight and obesity and chronic non-communicable diseases, but also generate healthy and sustainable conditions, including benefits for the environment.

In addition, it is important that there is a commitment not only from government control agencies, but also from the owners of school canteens, school directors, and parents, and for this, it is important that policies are also socialized in order to understand and effectively take the necessary actions at home and school for the well-being of children.

## Figures and Tables

**Table 1:** Study participants: health experts, school canteen owners, directors of schools, and parents.

Health Experts					
Nr.	Code	Sex	Province	Type of School	Zone
1	Expert1	Male	Azuay	n/a	n/a
2	Expert2	Male	Azuay	n/a	n/a
3	Expert3	Male	Cañar	n/a	n/a
4	Expert4	Male	Morona Santiago	n/a	n/a
5	Expert5	Female	Azuay	n/a	n/a
School Canteen Owners					
1	Owner1	Male	Azuay	Public	Rural
2	Owner2	Female	Azuay	Public	Urban
3	Owner3	Female	Cañar	Public	Rural
4	Owner4	Female	Morona Santiago	Public	Rural
5	Owner5	Male	Cañar	Private	Urban
6	Owner6	Female	Morona Santiago	Private	Urban
Parents					
1	Parent1	Female	Azuay	Public	Rural
2	Parent2	Male	Azuay	Private	Urban
3	Parent3	Female	Cañar	Private	Rural
4	Parent4	Female	Cañar	Private	Rural
5	Parent5	Female	Morona Santiago	Public	Urban
6	Parent6	Female	Morona Santiago	Public	Rural
7	Parent7	Male	Azuay	Private	Urban
Directors of Schools					
1	Director1	Female	Azuay	Public	Urban
2	Director2	Female	Azuay	Public	Urban
3	Director3	Male	Cañar	Public	Rural
4	Director4	Female	Azuay	Private	Rural
5	Director5	Female	Morona Santiago	Private	Urban
6	Director6	Male	Morona Santiago	Private	Rural

n/a: non-applicable.

## Data Availability

Due to the ethical and bioethical regulations of the University of Cuenca in relation to the people who were part of the study, the database cannot be socialized.
